# Role of Invertebrate Biological Origin in Chitin Nanocrystal’s Morphology, Chirality, and Self-Assembly

**DOI:** 10.1021/acs.langmuir.5c01167

**Published:** 2025-06-05

**Authors:** Murat Kaya, Kui Yu, Kine Østnes Hansen, Mohammed Al-dubai, Martin Vinther So̷rensen, Muhammad Mujtaba

**Affiliations:** † Department of Molecular Biology and Genetics, Faculty of Science and Letters, 52971 Istanbul Technical University, Istanbul 34469, Turkey; ‡ Yusuf Hamied Department of Chemistry, University of Cambridge, Lensfield Road, Cambridge CB2 1EW, U.K.; § Department of Bionanoscience Kavli Institute of Nanoscience, 2860 Delft University of Technology, Van der Maasweg 9, Delft 2629, HZ, The Netherlands; ∥ Marbio, UiT−The Arctic University of Norway, Breivika, Tromso̷ N-9037, Norway; ⊥ Natural History Museum of Denmark, 4321 University of Copenhagen, 2100 Copenhagen, Denmark; # VTT Technical Research Centre of Finland Ltd, P.O. Box 1000, Espoo FI 02044, Finland

## Abstract

The mention of chitin often evokes the Bouligand structure, which is a unique twisted configuration featuring a uniaxial planar organization of fibers. Although a large number of studies focused on Arthropoda, the architecture of chitin in many other invertebrate phyla remains largely unexplored. Herein, we unveil the distinctive architectures of chitin in both Arthropoda and Bryozoa, offering a comparative analysis of the morphological properties of native fibers and chitin nanocrystals sourced from these divergent organisms. In stark contrast to the Bouligand architecture prevalent in Arthropoda, Bryozoa exhibits a unique spiderweb-like arrangement of nanobundle structures, exclusive to this animal group. Bryozoan chitin nanofibers have a diameter smaller than those found among arthropods. After acid hydrolysis, the bryozoan nanocrystals are shorter and have a diameter smaller than those from arthropods. Although the chitin nanocrystals formed the chiral nematic phase, in the current study with the applied methodology, this was not the case with chitin nanocrystals from the studied bryozoan species. The unique chitin nanoarchitecture observed in Bryozoa could serve as an inspiration to produce advanced materials. Their smaller chitin nanocrystals can serve as a potential alternative to those of arthropods.

## Graphical abstract

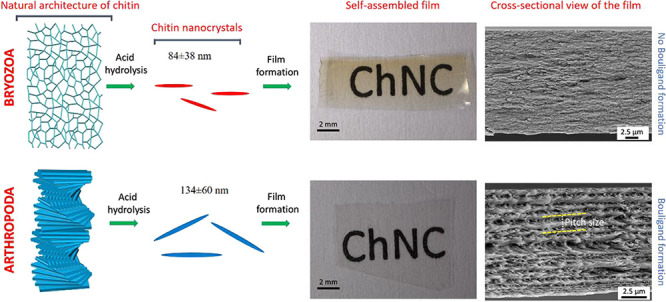

## Introduction

Bioinspired architectures are of great interest to many disciplines for the development of new technologies and industrial purposes such as superhydrophilic and superhydrophobic surface architecture,^1^ sensing and energy storage,^2^ flexible electronic devices,^3^ and micro/nanomotors.^4^ One of these natural architectures is the Bouligand structure of layered and rotating microcrystals, which is well-known for cellulose nanocrystals and chitin, with proteins and minerals found in arthropod cuticles.^5^ The mention of chitin often evokes the Bouligand structure found exclusively in arthropod shells. Chitin is arranged in a Bouligand structure, a unique twisted configuration.^6^ This layered and rotating microstructure, featuring a uniaxial planar organization of fibers, contributes to both the exoskeleton’s iridescence and its exceptional strength. It has been reported that this design functions to effectively dissipate impact energy and prevent crack propagation, providing the exoskeleton with exceptional resilience.^7^ Moreover, the Bouligand arrangement displays a lyotropic liquid crystalline phase with supramolecular chirality, a fundamental natural property.^8^ Chirality is an inherent feature in biological molecules like proteins, amino acids, and polysaccharides, and plays a critical role in defining both the function and potential applications of these materials.^9^ This serves as a crucial muse for engineering mechanically robust materials, which effectively mitigate outer fracture initiation and propagation across multiple directions.^10,11^ Thus, chitin is expected to be found in the Bouligand structure in most organisms, but this is not always the case because Arthropoda is only one of 31 invertebrate phyla. However, the structure of natural chitin is still left unexplored for numerous other invertebrate phyla, and we do not know how the chitin architecture in these creatures can be a source for nature-inspired materials.

The formation of rod-shaped chitin nanocrystals precipitated following the digestion of less ordered chitin domains under the rigorous conditions of boiling concentrated acid culminates in the self-assembly of solid films characterized by intricate Bouligand structures.^12^ However, earlier studies of the self-assembled chitin nanocrystals focused mostly on arthropods.^13–15^ Apart from Arthropoda, only a single recent study has shown that chitin nanocrystals produced from the fungi kingdom turn into films of self-assembled Bouligand structure.^14^ Both arthropods and fungi produce rod-shaped chitin nanocrystals consisting of α-chitin. On the contrary, Jung, Kim, and Park^16^ reported that acid hydrolysis of species from the animal phylum Mollusca produces more rounded nanocrystal particles made of β-chitin. Observation of round nanoparticles from Mollusca might be an expected result because β-chitin from Mollusca (excluding diatoms and tube worms) has lower crystallinity and lower thermal stability than α-chitin. The authors reported that in β-chitin, the abundance of amorphous regions interspersed with individual crystalline areas could lead to the formation of more circular-shaped particles.^16^ The decreased crystallinity and thermal stability of β-chitin, compared to α-chitin, can be attributed to its lower N-acetyl content.

Now we are wondering: can rod-shaped chitin nanocrystals be produced from α-chitin belonging to other living phyla, and if it is produced, can the nanocrystals of these chitins turn into Bouligand-structured films by chirality, as observed in Arthropoda and Fungi?

The present study focuses on the phylum Bryozoa, which accommodates close to 6,000 species, mostly distributed in marine habitats. Only a single study isolated chitin from a freshwater bryozoan species (Plumatella repens) and made a characterization.^17^ However, no study has shown the natural architecture or nanocrystal production of chitin in this phylum. To represent the arctopoda, the sea spider, which has never been investigated for chitin purposes, and the mealworm, which is phylogenetically far from the sea spiders, are included.

The two selected target phyla, Arthropoda and Bryozoa, were chosen for the study because they represent two very distantly related groups within the large invertebrate clade Protostomia. The protostomes, which accommodate >99% of all living species, can be divided into three main lineages:^18^ The Ecdysozoa—a diverse group of animals with hard cuticle, which includes the Arthropoda, but also groups like Nematoda and Tardigrada; the Gnathifera—mostly microscopic animals with hard jaw parts, such as Rotifera, but also the macroscopic Chaetognatha; and finally the Lophotrochozoa—another highly diverse clade that accommodates the Bryozoa, but also well-known phyla like Mollusca and Annelida. Recent molecular clock studies suggest that the last common ancestor of the two selected phyla dates between 580 and 636 million years ago.^19^

As a hypothesis, this study posits that chitin extracted from the Bryozoa phylum will exhibit a distinct architectural arrangement compared to Arthropoda, reflecting evolutionary divergence, and subsequent acid hydrolysis will yield nanocrystals with differing morphologies, showing varied chiral behaviors potentially resulting in unique Bouligand-structured films characteristic of each phylum.

This study uncovers the natural architecture of chitin in Bryozoa and draws comparisons with chitin from two distinct arthropod species. As a result, a novel chitin architecture emerges, potentially offering innovative material design inspiration. Furthermore, we isolated chitin from these sources and subjected it to acid hydrolysis to produce nanocrystals, revealing variations across different phyla. Additionally, we conducted a comparative examination of the chiral characteristics of these nanocrystals within each phylum.

## Materials and Chemicals

The fresh sample of Securiflustra securifrons belonging to the phylum Bryozoa was collected from off the coast of Ro̷nnbeck Islands, East Spitzbergen, Norway (Latitude: 79.0273, Longitude: 20.8618) with a triangular dredge from 48 m deep on 09.10.2011. The samples were sorted onboard the ship and stored at −23 °C in the dark. Also, the sample was transferred from Norway to the U.K. on dry ice. Then the sample was dried in an oven at 40 °C for 1 week, and around 80 g of sample was used for this study. For Arthropoda, Pycnogonum litorale, collected in Denmark, was provided by the Natural History Museum of Denmark. The mealworm sample (belonging to the species Tenebrio molitor) was purchased from the local market directly. Hydrochloric acid (HCl), sodium hydroxide (NaOH), and hydrogen peroxide (H_2_O_2_) used in the study were obtained from Sigma-Aldrich.

### Natural Architecture of the Chitin

To determine the natural architecture of chitin in the organisms, the samples (about 50 mg for each) were subjected to mild acid and base while keeping the original shape of the materials. First, to remove the minerals, the samples were treated with 40 mL of 1 M HCl solution at room temperature for 12 h. Afterward, the samples were washed in a beaker by continuously adding and changing Milli-Q water until reaching neutral pH. Second, to remove the protein, the wet samples (without drying) were treated with 30 mL of 1 M NaOH solution at 40 °C for 12 h in a reflux system with gentle mixing. The intact samples were subsequently vacuum-filtered through nitrocellulose filter paper (pore sizes: 0.8 μm) by adding Milli-Q water until reaching neutral pH. For each material, half of the samples were freeze-dried, and the other half was dried at room temperature. The dried samples were transferred gently by sharp tweezers onto conductive carbon tape on aluminum stubs. Then the surface of the samples was sputter-coated with 10 nm thick Au/Pd by using a Quorum Q150T ES. The natural architecture of chitin for each sample was demonstrated with the images taken from the cross-section and both surfaces of the samples using the TESCAN MIRA3 FEG-SEM device.

### Chitin Isolation

Since the mineral and protein contents of the studied organisms are different, we applied chitin isolation methods separately for each phylum.

In the preisolation experiments with a small number of samples, it was observed that the mineral and protein contents of the Bryozoa samples were high, but the chitin content was also quite low. Isolation of the chitin from Bryozoa started with 50 g of a dry sample. For demineralization, the sample was treated in a 2 M 500 mL of HCl solution at room temperature for 10 days because the emergence of air bubbles from the solution ceased after 9 days. The samples were then recovered by centrifugation at 10,000 rpm and 4 °C for 3 replications. To reach neutral pH, the sample was dialyzed (Regenerated Cellulose dialysis tubing, MWCO 12–14 kDa, Scientific Laboratory Supplies) against Milli-Q water for 8 days. During the dialysis, Milli-Q water was changed every 10 h. The demineralized sample was treated for 16 h in 2 M 200 mL of NaOH solution in a reflux system at 90 °C with stirring. To achieve neutral pH, after demineralization, the samples were first centrifuged and then dialyzed against Milli-Q water for 12 days. The sample was then decolorized in 100 mL of 5% H_2_O_2_ solution at 50 °C for 2 h in a reflux system with stirring. Similarly, the sample was centrifuged 3 times and then dialyzed against Milli-Q water for 8 days. Then the samples were dried in an oven at 50 °C for 5 days.

Before initiating the isolation, both arthropod species were washed with Milli-Q water and left to dry for a week at 50 °C. The same method was applied for both species. 5 g of intact samples (without powdering) was treated in 2 M 100 mL HCl solution in a reflux system at 40 °C for 6 h. Because the sample size is quite large, the used acid was removed by using a sieve (mesh size: around 200 μm), and neutralized samples were obtained. Then the samples were refluxed in 3 M 100 mL of NaOH solution at 90 °C for 18 h. The intact samples were rinsed until they reached neutral pH with Milli-Q water by using the sieve for 9 h. The samples were decolorized with H_2_O_2_ using the same method applied to the Bryozoa sample. The obtained intact arthropod chitin samples were dried at room temperature and made ready for analysis and further experiments.

### Chitin Hydrolysis

Different amounts of chitin were used for the acid hydrolysis for each source. For all chitin isolates, the method applied by Narkevicius, Parker, Ferrer-Orri, Parton, Lu, van de Kerkhof, Frka-Petesic, and Vignolini^14^ was followed with minor modifications. For hydrolysis of chitin, 3 M HCl (6 mL solution for 100 mg dry chitin) at 105 °C for 4 h was used. The initial amounts of dry chitin isolates for hydrolysis were 400 mg for Bryozoa, 200 mg for sea spiders, and 500 mg for mealworm. After 4 h, the reaction was quenched by double dilution with ice-cold water. The hydrolyzed samples were recovered by centrifugation at 25,000 g for 30 min at 4 °C and dispersed in Milli-Q water. This centrifugation process was repeated 3 times by adding Milli-Q water each time. After centrifugation, the samples were dialyzed against Milli-Q water for 6 days until reaching neutral pH. The samples at neutral pH were dialyzed against 0.6 × 10^–3^ M HCl solution until the conductivity in the dialysis bath stopped changing overnight. Subsequently, the samples were transferred from the dialysis bags to Falcon tubes and suspended by vortexing. The suspensions for each sample were tip sonicated for 6.75 s mL^–1^, at 1 wt % using 30% amplitude (10:15 pulse for 102 s) under an ice bath using Fischer Sonic Dismembrator, 500 W, and filtered using 8.0 and 0.8 μm nitrocellulose filter paper, Millipore, Germany. All the samples were concentrated to 2 ± 0.18 wt % using a rotary evaporator.

### Production of Films from Chitin Nanocrystals

The suspensions adjusted to 2.0 wt % were poured into Petri dishes (diameter 35 mm) and dried at room temperature for 3 days. Bryozoa and sea spider films were adhered to the bottom of the Petri dish, while the mealworm film was easily removed from the Petri dish by peeling. Sea spider and mealworm films were found to be more fragile than bryozoan films during the removal and photographing of the films from the Petri dish.

### Scanning Electron Microscopy

The samples were coated with Au/Pd (10 nm) with a Quorum Q150T ES. The images were taken with a TESCAN MIRA3 FEG-SEM scanning electron microscope. Bryozoa samples that were dissected under a Keyence Microscope (VHX-7000, Keyence, Japan) into 1 × 1 mm pieces were taken into Karnovsky’s fixative (2% paraformaldehyde, 2.5% glutaraldehyde, and 0.1 M buffer) for 1 week. The bryozoan pieces were stained with osmium tetraxide for 2 h. After that, the pieces were taken through a graduated ethanol–resin series before embedding in Epon 814 resin, with DMP added for accelerating before drying at room air pressure at 60 °C. The resin block samples were polymerized for 24 h under a vacuum at 60 °C. A Leica UCT ultramicrotome was used to cut ultrathin sections (approximately 100 nm) with a diamond knife. The sections were illustrated using an FEI Verios 460 scanning electron microscope. The cross-section of chitin nanocrystals (ChNCs) solid films was analyzed using SEM.

### Transmission Electron Microscopy

The same method was followed for both chitin nanofibers and chitin nanocrystals. 25 μL of the suspended chitin nanofibers (ChNFs) and chitin nanocrystals (ChNCs) (0.006 wt %) were pipetted onto a carbon-coated copper grid (afterglow discharging) and left for 40 s. Subsequently, the liquid was removed using tiny pieces of filter paper, followed by staining with 25 μL of uranyl acetate (aqueous, 2.00 wt %) for 40 s. The samples on the grids were left to dry overnight at room temperature before imaging. Then the images were taken using a Talos F200X G2 microscope (FEI) operating at 200 kV and a CCD camera. The length, width, and aspect ratios of 100 nanofibers and nanocrystals for each sample were measured by using ImageJ software.

### Fourier Transform Infrared Spectroscopy

Infrared spectra of the isolated chitins and the films were recorded in the range 4000–700 cm^–1^ with 64 repeats at a resolution of 4 cm^–1^ using the 100 ATR PerkinElmer Spectrometer.

### Thermogravimetric Analysis

Thermogravimetric analysis was conducted to determine the maximum degradation temperatures, moisture, and ash contents of ChNFs and ChNCs. The samples were analyzed by using an EXSTAR S11 7300 under a nitrogen atmosphere (3 mL/min purge rate) by heating from 30 to 700 °C using aluminum crucibles at a constant temperature of 10 °C min^–1^.

### XRD

Powder X-ray diffraction data of ChNFs and ChNCs were collected on a Malvern Panalytical Empyrean instrument, equipped with an X’celerator Scientific detector using nonmonochromated CuKα radiation (λ = 1.5418 Å). The sample was placed on a glass sample holder and measured in a reflection geometry with sample spinning. The data were collected at room temperature over a 2θ range of 5–40 °, with an effective step size of 0.01° and a total collection time of 60 min.

### Elemental Analysis

CHN combustion analysis was performed on an Exeter Analytical, Inc. CE-440 Elemental Analyzer with a combustion temperature of 975 °C. Approximately 2 mg was taken for each sample, and the average was taken by measuring twice.

### Digital Microscope

A Keyence Microscope (VHX-7000, Keyence, Japan) was used to obtain images of the original samples after acid and base treatments.

### Dynamic Light Scattering and Zeta Potential

The size distributions and surface charges of the ChNCs in the suspensions (0.1 wt %) in water were measured at pH 3 and 22 °C with a zeta potential analyzer (Zetasizer 3000, Malvern Panalytical). The size distribution of the nanocrystals was obtained by measuring the ζ-potential (the Smoluchowski correction function) with three repetitions and 50 runs each. Before each measurement, the suspension solution was gently shaken to obtain a uniform size distribution of ChNCs.

## Results and Discussion

To get an overview of the appearance difference, optical images of Bryozoa (Figure 1a), sea spiders (Figure 1b), and mealworms (Figure 1c) were obtained.

**Figure 1:**
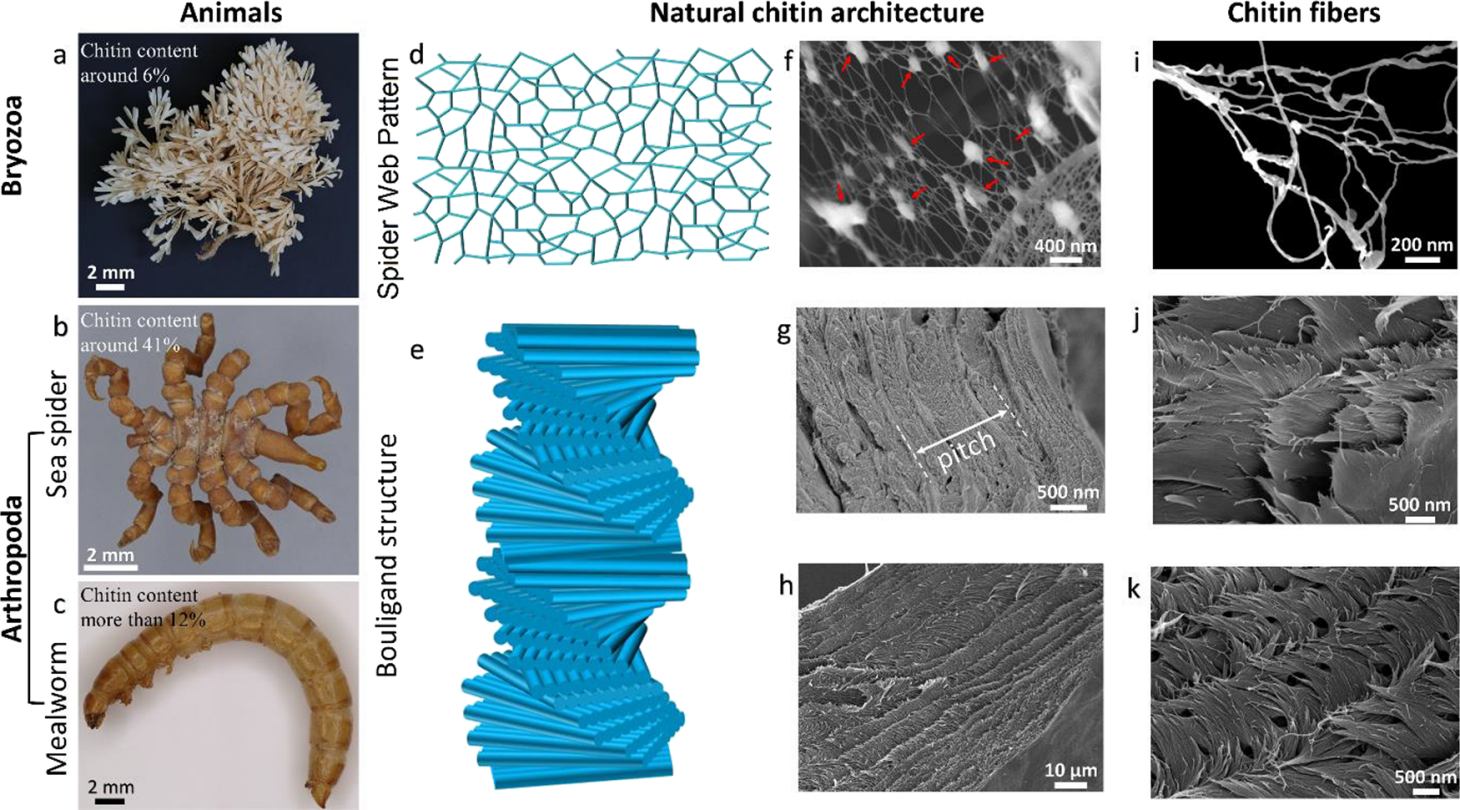
Studied groups and species for chitin isolation: (a) Bryozoa (Securiflustra securifrons), (b) sea spider (Pycnogonum litorale), and (c) mealworm (Tenebrio molitor). (d) Illustration of natural architecture (spider web pattern) of chitin observed in Bryozoa and (e) known Bouligand architecture in Arthropoda (sea spider and mealworm cuticle). SEM images of parts (f) and (i) demonstrate the spider web pattern in Bryozoa after mild acid and base treatment to the bryozoan sample. The red arrows indicate the presence of residual protein following mild acid and base treatment. The reason behind using mild acid and base treatment during the extraction process is to protect the natural/native architecture of bryozoan chitin. (g,j) Bouligand structure in sea spider and (h,k) Bouligand structure in mealworm. Additional SEM images with lower magnifications have been provided in Figure S3.

### Natural Architecture of Chitin in Bryozoa and Arthropoda

The natural architecture of chitin in Bryozoa and Arthropoda, chitin nanobundles, and isolated chitins is shown in Figure 1. The detailed structure of the bryozoan sample revealed (by SEM) that the natural architecture of chitin is a spider web-like structure (Figure 1d,f,i). Different from bryozoan, Bouligand architecture was observed in both studied arthropod species (Figure 1e,g,h,j,k), which is as expected and has been widely studied.^11^ As illustrated in the figure (Figure 1j,k), the discontinuous short nanofibers also represent the classical Bouligand architecture.^11^

Ultrathin sections (around 100 nm) cut from the bryozoa sample using an ultramicrotome are shown using SEM (Figure 2a) and TEM (Figure 2b,c). Cross-sectional images show that Bryozoa consists of four different layers: layers A–D (Figure 2b,c). Layers A, B, and C are located equally and parallel on both sides, while Layer D is located in the middle. Layers A and C were observed to be rich in minerals (densely) and protein (small amount); however, no chitin was detected in these layers. The presence of chitin along with minerals and protein was observed in the layers called B and D. Thanks to the demineralization of layer A with acid (HCl) application, the surface morphology of chitin in layer B was revealed, while the nanostructure of chitin in layer D could not be determined. The reason why chitin is not observed in layer D is probably due to the lower amounts of chitin in this layer. Therefore, mild acid and base applications led to the disintegration of the 3-dimensional structure of Layer D.

**Figure 2:**
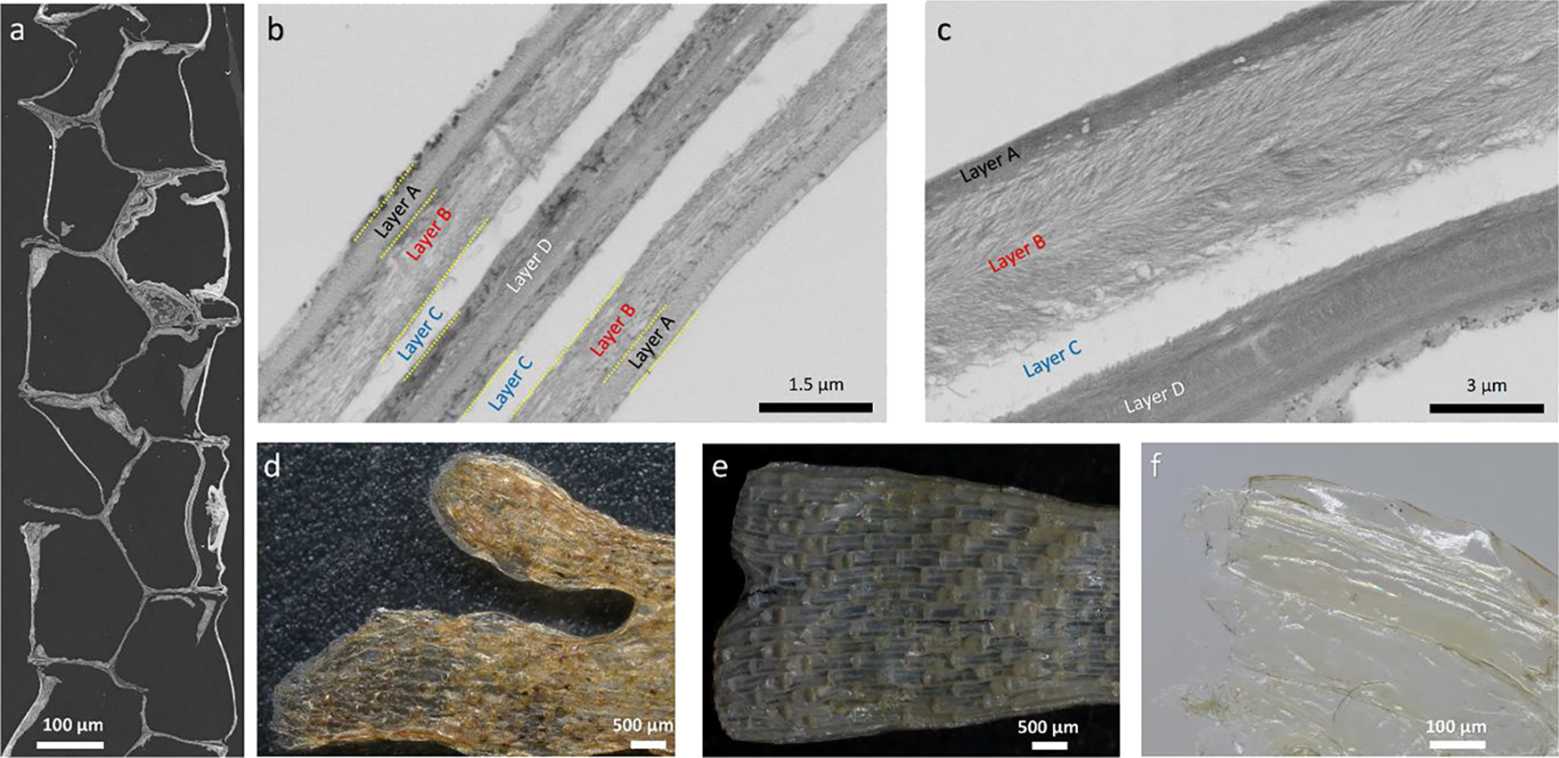
(a) SEM images of the ultrathin bryozoan section (around 100 nm) cut by an ultramicrotome. (b,c) Cross-sectional TEM images from the original Bryozoa sample. The marked layers A and C were observed to be rich in minerals (densely) and protein (small amount); however, no chitin was detected in these layers. B was rich in chitin (the web like structure in layer B is provided in Figure[Fig fig1]f,i, SEM images), and D was rich in minerals and lower amounts of chitin, (d) bryozoa sample before sodium-hydroxide treatment, (e) sodium hydroxide-treated bryozoa sample, and (f) obtained bryozoan film after acid and base treatments.

As a result of mild acid treatment, the original shape of the Bryozoa specimen shrinks (Figure 2d), which indicates the high proportion of minerals in Layer A and C (Figure 2b,c), and these minerals play an important role in supporting the tissue to avoid tissue shrinkage. After a small amount of base was applied to the original sample, the proteins were removed from the structure, but minor changes were observed in their original form (Figure 2e), indicating that minerals are more effective than proteins in maintaining the three-dimensional structure in the studied bryozoan species. After mild acid and base treatments, a thin and transparent film (Figure 2f) was formed due to the removal of a large amount of minerals and proteins from the structure. Ultrathin sections of a sea spider specimen, representing the phylum Arthropoda, were prepared using microtomy, and the resulting TEM image clearly reveals the natural Bouligand structure (Figure S1).

The isolated chitins from the bryozoa and arthropod species are shown in Figure 3a–c. Chitin is found in the cuticles as nanobundles (Figure 3d–f) consisting of tens of nanofibers (Figure 3g–i). The diameters of ChNFs differ according to the studied organisms. It has been observed that bryozoan chitin nanofibers (BChNFs) (3.97 ± 0.99 nm) are thinner than sea spider (SChNFs) (4.86 ± 1.28 nm) and mealworm chitin nanofibers (MChNFs) (8.92 ± 3.1 nm) in diameter (Figure 3j–l). According to the literature, the thickness of α-chitin nanofibers isolated from insect cuticles varies between 3 and 7 nm (average 6.2 nm),^20^ the thickness of chitin nanofibers isolated from crab shells varies between 10 and 20 nm,^21^ while the thickness of β-chitin nanofibers isolated from squid pen is 3–4 nm.^22^ Overall, the thickness of the chitin nanofibers isolated from Bryozoa is closer to that of β-chitin isolated from molluscs compared to that of α-chitin in arthropods.

**Figure 3:**
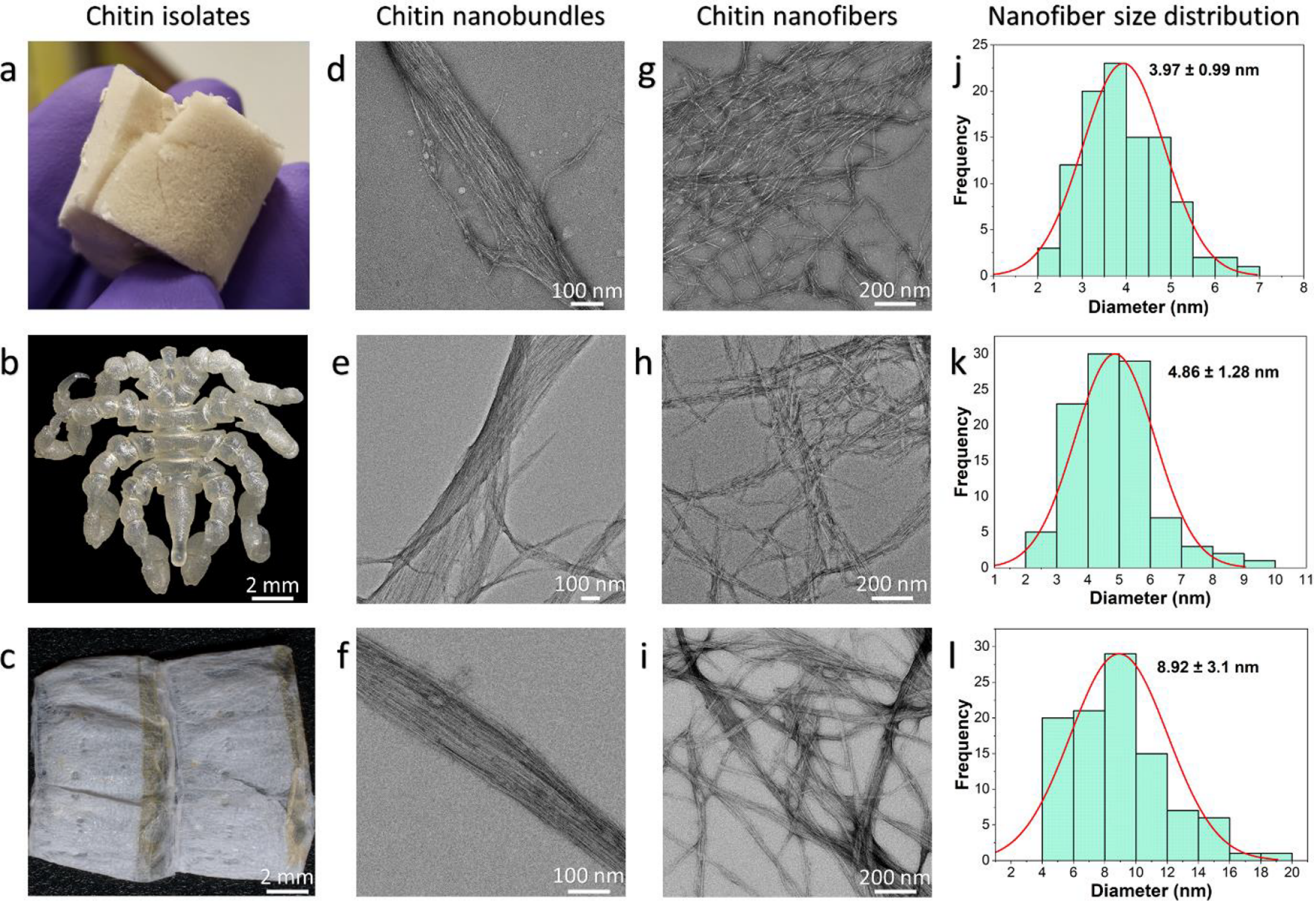
Obtained chitin samples from (a) bryozoa, (b) sea spider, and (c) mealworm. (d–f) TEM images of nanobundles of chitin from (d) Bryozoa, (e) sea spider, and (f) mealworm. TEM images of chitin nanofibers were obtained from (g) Bryozoa, (h) sea spider, and (i) mealworm. Size distribution of chitin nanofibers based on TEM images obtained from (j) Bryozoa, (k) sea spider, and (l) mealworm. More images for the chitin nanobundles and nanofibers are provided in the Supplementary data (Figure S3).

### Characterization of Chitin Isolates and Chitin Nanocrystals

FTIR spectra of isolated chitin and ChNCs are shown in Figure 4a. The clear observation of amide I, II, and III bands, which are characteristic of chitin, indicates that the chitin was successfully obtained and that the isolated materials are of high purity.^23^ Splitting the amide I band into two (around 1650 and 1620 cm^–1^), like other arthropod chitins, reveals that Bryozoa also contains α-chitin.^24^ Especially for bryozoa chitin (BCh), the sharpness of the peaks increases because of nanocrystal production. This result shows that BCh has more amorphous parts than arthropod chitins, and more crystalline fragments are formed because of the digestion of these parts by acid hydrolysis.

**Figure 4:**
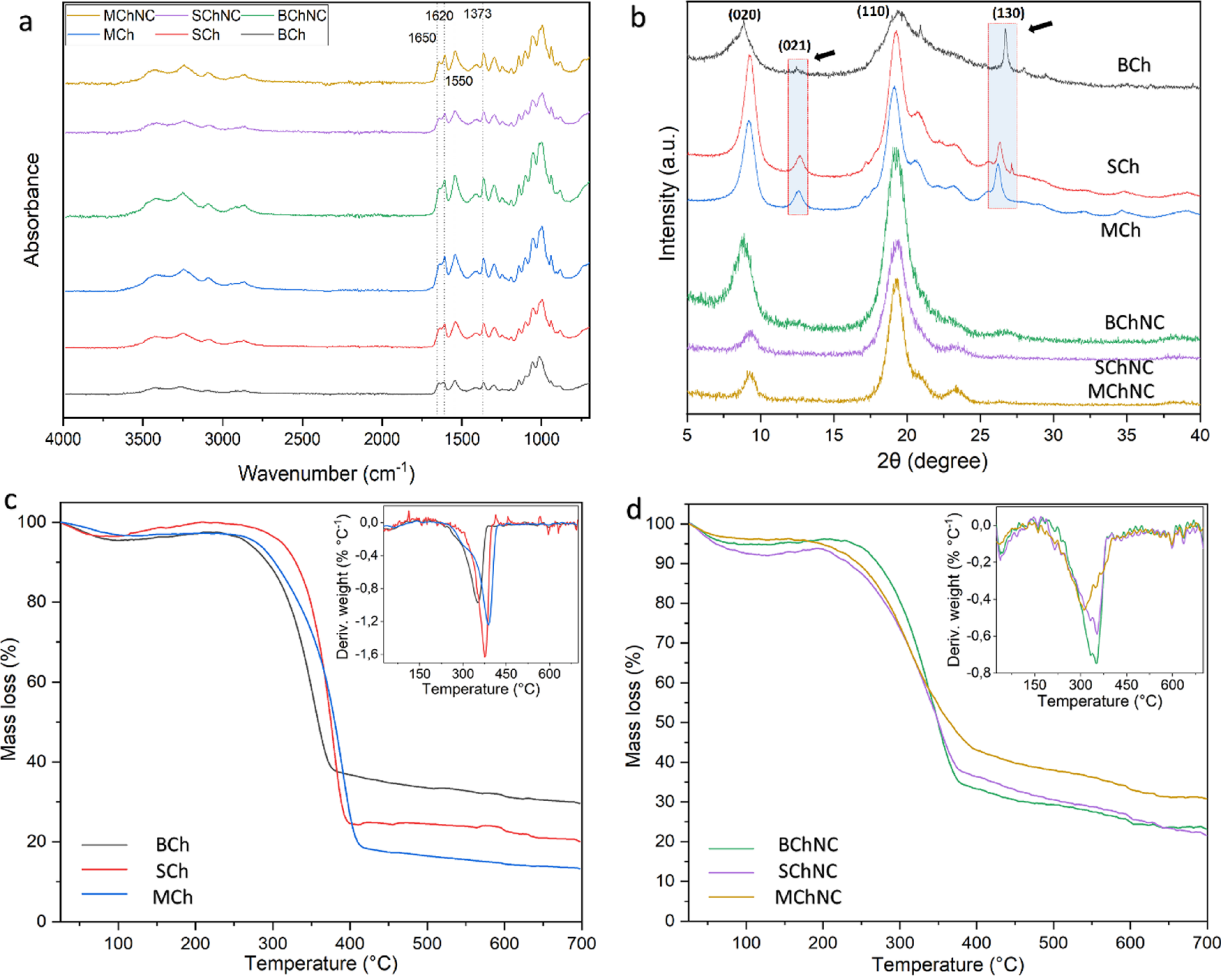
Analysis results of chitin isolates and chitin nanocrystals obtained from Bryozoa, sea spider, and mealworm. (a) Fourier transform infrared spectroscopy (FTIR) and (b) X-ray diffraction (XRD). After acid hydrolysis, the intensity of the peaks shown by the arrows has been decreased due to a reduction in crystal size and changes in preferential orientation of the chitin in the sample holder. (c) Thermogravimetric analysis (TGA) and (d) derivative thermogravimetric analysis (DTG).

For XRD analysis of chitin isolates, two sharp peaks at 9° and 19° and weak peaks at 13°, 21°, 23°, and 26° were observed (Figure 4b), which are characteristic of chitin.^24^ After the acid hydrolysis of chitins, the peaks around 13° and 26° (shown with arrow in Figure 4b) seem to weaken; this can be attributed to the reduction in crystal size and change in preparation orientation (alignment or arrangement of crystalline domains) during acid hydrolysis where the chain axis was in-plane with the sample holder plane. This geometry in the reflection mode should enhance the equatorial reflection intensities and weaken those from the higher layer lines, including (021) at 13° and (130) at 26° (for Miller index and *d*-spacing, see Table S3).

The thermal properties of the obtained chitin and chitin nanocrystals were investigated (Figure 4c,d), and the TGA results are summarized in Table S1. ChNCs have been noted to absorb more water than chitin isolates. It is observed that the maximum decomposition temperature of BCh (357.5 °C) is lower than that of the arthropod chitin isolates. A maximum decomposition temperature of over 350 °C indicates that chitin is in alpha form, whereas low decomposition indicates that chitin is in beta form.^24^ Considering this result, BCh is in the alpha form. When we look at the literature data, it appears that BCh has lower thermal stability than arthropod chitin.^23^ Surprisingly, chitin becomes less thermally stable when it is converted into nanocrystals. This is contrary to what would be expected because the digestion of the amorphous parts of chitin should yield more stable chitin parts. The reduced thermal stability of chitin nanocrystals may be caused by a higher surface area or altered surface charges.

The % C, H, and N contents of the chitin samples were determined by elemental analysis, and the degrees of acetylation (DA) were calculated (Table S2). Accordingly, the DA values of bryozoa, sea spider, and mealworm chitins were calculated as 98.35 ± 1.06, 99.1 ± 0.42, and 85.3 ± 0.66, respectively. According to these results, bryozoa and sea spider chitins are purer than mealworm chitin.

### Chitin Nanocrystals

The same method was used for the production of nanocrystals from all of the chitin samples. The produced nanocrystals were imaged with TEM (Figure 5a–c), and the lengths, thicknesses, and aspect ratios of the nanocrystals were measured based on these TEM images (Figure 5d–g). The lengths and widths of BChNCs (84 ± 38, 6 ± 2 nm) were considerably smaller than those of SChNCs (134 ± 60, 8 ± 2 nm) and MChNCs (174 ± 90, 11 ± 5 nm). Aspect ratios were calculated as 13 for BChNCs, 18.8 for SChNCs, and 18.9 for MChNCs. The nanocrystal size distributions were also confirmed by DLS analysis, with results similar to those measured with TEM images. DLS analysis revealed that BChNCs were smaller in length (120 ± 76 nm) than SChNCs (186 ± 87 nm) and MChNCs (224 ± 86 nm). Positive ζ-potentials were recorded for all of the chitin nanocrystals at pH 3. The ζ-potentials were measured as 26 ± 1, 27 ± 1, and 37 ± 2 mV for BChNC, SChNC, and MChNC, respectively, which are in line with the literature data.^15,25^

**Figure 5:**
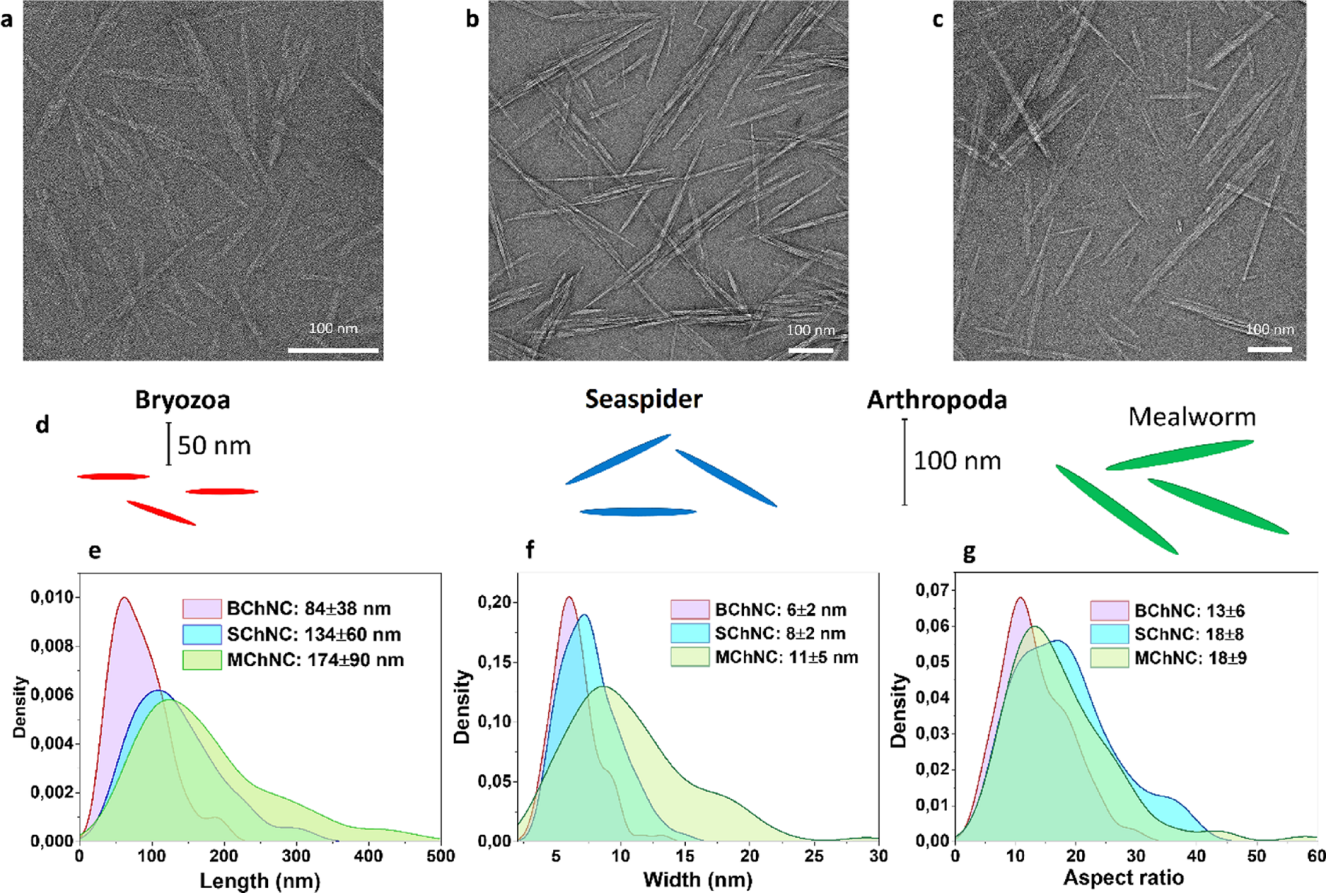
TEM images of chitin nanocrystals produced from (a) Bryozoa, (b) sea spider, and (c) mealworm. (d) Representation of the sizes of the obtained nanocrystals depending on the studied organisms. (e) Length, (f) width, and (g) aspect ratio of the chitin nanocrystals according to the studied groups. For chitin nanocrystals, more images are provided in Figures S5–S7.

In previous studies, it has been observed that the size of chitin nanocrystals produced by different methods from various arthropod taxa and a mushroom species (Agaricus bisporus) varies between 150 and 500 nm in length and between 8 and 21 nm in width.^14,15,26^ Considering both TEM images and DLS results, BChNCs are considerably shorter and thinner than chitin nanocrystals from arthropod species in this study, as well as from other arthropod taxa and fungal chitin nanocrystals reported in the literature.

Although rounded nanocrystals were reported from species of Mollusca,^16^ rod-shaped nanocrystals have also been observed from bryozoans, as also observed in other α-chitins from Arthropoda and the kingdom fungi. In this case, it seems likely that rod-shaped nanocrystals can be obtained from the α-chitin form. However, as observed in BChNCs in this study, the length/width/aspect ratio of these rod-shaped nanocrystals may also vary according to the phyla.

### Chiral Properties of Chitin Nanocrystals

The Bouligand structure is an exquisite example of hierarchical organization, characterized by fibers aligned in a specific direction that helicoidally rotate with a defined handedness—left-handed in the case of chitin. This unique arrangement is well-known in the soft matter community as a “cholesteric” or “chiral nematic” structure. Unlike simple fiber alignment caused by external forces such as shear, the Bouligand structure arises from the intrinsic properties of the fibers, demonstrating their remarkable capacity for self-assembly. The formation of a Bouligand structure exemplifies a higher degree of self-organization where the fibers autonomously initiate and maintain a helicoidal arrangement. This ability for self-assembly is crucial for understanding the presence of such intricate structures in biological materials like chitin.

Self-assembly is a fundamental phenomenon observed across various scales in nature, where components spontaneously organize into ordered structures without an external direction. Although fibers tend to align under shear flow universally, this process does not constitute self-assembly since it relies on external influences. In contrast, genuine self-assembly involves the spontaneous reorganization of fibers from an isotropic state into a nematic alignment, a concept first detailed by Lars Onsager in his seminal work on nonchiral rods.^27^ The Bouligand structure and its formation through self-assembly highlight the sophisticated mechanisms by which nature achieves complex and functional architectures, providing profound insights into the principles that govern the organization of matter across different scales.

The chiral properties of chitin nanocrystals produced from Bryozoa and two different arthropod species were investigated. After pouring the aqueous suspension containing 2 wt % of nanocrystals into the Petri dish and drying for 3 days at room temperature, transparent films were obtained from all the nanocrystal samples (Figure 6a–c). Sea spider chitin nanocrystal film (SChNCF) (Figure 6b) and mealworm chitin nanocrystal film (MChNCF) (Figure 6c) were fully transparent, while bryozoan chitin nanocrystal film (BChNCF) (Figure 6a) showed a slightly yellowish color. When cross sections of the films were imaged with SEM, the Bouligand structure was observed in the arthropod films (Figure 6f–i). However, no Bouligand formation occurred in the films obtained from chitin nanocrystals of the studied bryozoan species (Figure 6d,e). Although the surface charges of both BChNC and SChNC are close, a chiral nematic form was observed in SChNC, while it was not observed in BChNC. For the BChNC, we increased the concentration of the aqueous suspension containing 2 wt % of nanocrystals to 3, 4, and 5%, and then filled in capillary tubes; however, we did not see any fingerprint pattern, which is characteristic of the chiral nematic phase (Figure S2).

**Figure 6:**
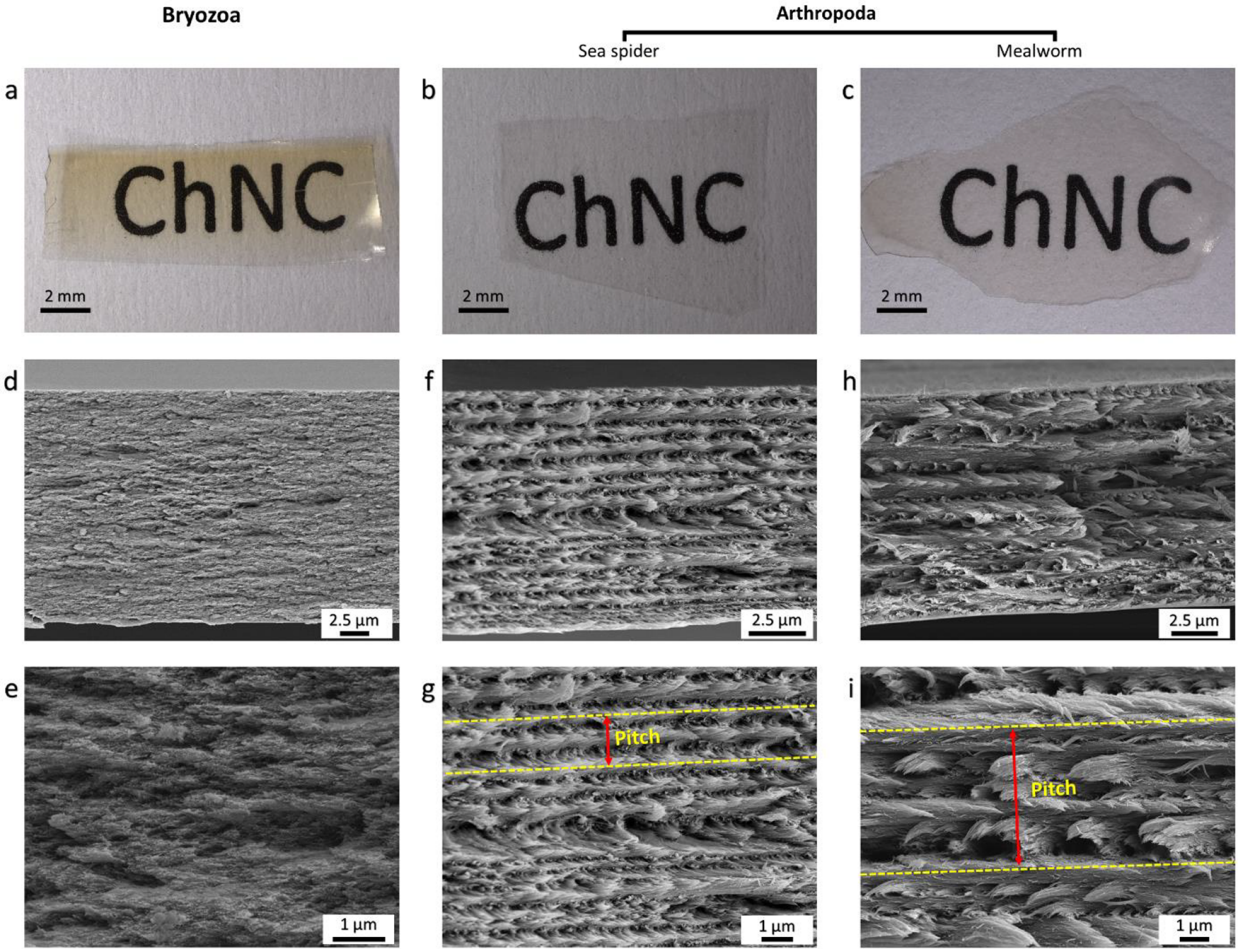
Films from (a) bryozoa, (b) sea spider, and (c) mealworm. (d,e) Cross-section of BChNCF (no chirality observed), (f,g) cross-section of SChNCF, and (h,i) cross-section of MChNCF. The length between the dashed yellow lines indicates the pitch size in the images (g,i).

Although both sea spider and mealworms are arthropods, the pitch sizes of the SChNCF (1.23 ± 0.33) (Figure 6g) are about two times smaller than MChNCF (2.59 ± 0.83) (Figures 6i and S6). According to Narkevicius et al.,^15^ this difference in pitch sizes could be due to the surface charge of nanocrystals, sizes of the produced nanocrystals, or the source of the organism, as differences were recorded between shrimp and fungal chitin nanocrystal chirality.^14^ There could be several factors contributing to the absence of chirality in bryozoan chitin nanocrystals (BChNCs) to form a Bouligand structure. For instance, BChNCs may necessitate different surface charge, concentration, or temperature conditions to initiate chirality compared to their Arthropod counterparts. The diminutive dimensions of BChNCs might also impede their chiral process. Additionally, if chitin does not naturally exhibit a Bouligand structure within the organism, then the resulting nanocrystals may similarly lack this feature. In contrast to our observations in the studied bryozoan species, chitin in Arthropoda naturally presents as a Bouligand structure, with chitin nanocrystals from Arthropoda demonstrating chiral capabilities mirroring their original architecture in living organisms. Another possible reason could be the adsorbed molecules on the chitin surface that could inhibit or promote the twisting of ChNCs. More plausibly, the presence of numerous chiral and twisted aggregates—abundant in tissues originally exhibiting a twisted structure—might be essential for the formation of a cholesteric suspension of ChNCs. In contrast, BChNCs lack these chiral aggregates, thereby only forming nonchiral, nematic-like structures. Parton et al.^28^ suggested the role of chiral aggregates in this context, although they did not discuss the origin of these aggregates. The apparent discrepancy between the chiral behavior of bryozoan and arthropod chitin nanocrystals prompts inquiry. However, it is crucial to emphasize that the anatomical structures of the arthropod cuticles and chitinous bryozoan exoskeletons are fundamentally distinct. While arthropod cuticles are rigid and nongrowing, necessitating periodic shedding and replacement during growth, bryozoan exoskeletons form protective compartments for zooids and interconnected tube-like systems within colonies. While this distinction does not fully explain the absence of chiral chitin nanocrystals in bryozoans, it underscores the functional divergence between arthropod and bryozoan exoskeletons. As suggested by a previous study,^15^ future investigations should comprehensively explore the chiral properties of BChNCs by systematically varying multiple parameters.

These results highlight the significance of broadening chitin studies beyond the confines of Arthropoda to encompass diverse invertebrate phyla, such as bryozoa. By elucidating the distinct nanoarchitectures of chitin in these organisms, we uncover insights with profound implications for material architecture and biomimicry. The unique spiderweb-like nanobundle structures found in bryozoa present a compelling avenue for innovation, offering a fresh perspective and potential alternative to the traditional Bouligand structure observed in Arthropoda. These results not only expand our understanding of chitin diversity but also pave the way for the development of novel materials with tailored properties and applications. In a rapidly evolving landscape of biomaterials research, the exploration of chitin’s diverse nanoarchitectures holds immense relevance for advancing material science and inspiring the next generation of biomimetic innovations.

It is established in the literature that molecular chirality exists in chitin, similar to cellulose; however, whether this molecular chirality is sufficient to induce ChNCs to assemble in a chiral configuration remains uncertain. Our results indicate that BChNCs derived from bryozoa, where chitin is not inherently chirally organized, do not exhibit chiral packing behavior. Conversely, SChNCs and MChNCs typically extracted from naturally chiral Arthropoda demonstrate a propensity for chiral arrangement. This observation suggests that the inclination of ChNCs to form chiral assemblies may not be an intrinsic characteristic of chitin itself, but rather an inherited property from the chiral or nonchiral nature of the source tissue.

Simulations of both cellulose and chitin nanocrystals have demonstrated that a certain degree of spontaneous twist in individual nanocrystals is anticipated, depending on the molecular arrangement within the crystal structure.^29^ This intrinsic twisting of nanocrystals is significant, as it could drive the chiral packing of nanocrystals. The observed discrepancy between simulations, which predict a spontaneous twist, and our empirical data, where BChNCs do not form a cholesteric phase, is intriguing. This discrepancy might provide insight into the underlying factors that influence the chiral or achiral behavior of chitin.

## Conclusions

In summary, we show that the chitin of bryozoans exhibited a spider web pattern, unlike the Bouligand structure in Arthropoda. We consider this architecture to be important in fabricating a new product based on chitin going forward. We determined that the thickness of chitin nanofibers obtained from bryozoa was smaller than that of Arthropoda. This result revealed that the thickness of the chitin nanofibers would vary considerably, according to the animal phylum. It was revealed that chitin nanocrystals produced from bryozoans were shorter and thinner than nanocrystals of arthropods. In this way, it was observed that the production of smaller rod-shaped nanocrystals could be produced from the Bryozoa phylum, unlike the chitin nanocrystals in Arthropoda. The desired smaller sizes of chitin nanocrystals produced from bryozoans and introduced in materials science can offer potential future applications in various biomedical fields (subject to further studies and results). Although the chitin nanocrystals produced from the Arthropoda exhibit chirality, it has been concluded that the chirality of the chitin nanocrystals obtained from different phyla may not be possible or different processes could be needed. This observation suggests that the inclination of ChNCs to form chiral assemblies may not be an intrinsic characteristic of chitin itself, but rather an inherited property from the chiral or nonchiral nature of the source tissue. Our work suggests that the chiral organization of the source tissue from which ChNCs are extracted is crucial for producing chiral suspensions and films. Understanding these mechanisms further can elucidate the conditions under which ChNCs adopt chiral configurations, ultimately advancing the field of nanomaterials and their applications in biomimetic structures.

## Supplementary Material



## Notes

Thermal and elemental analysis of chitin isolates from Bryozoa, sea spider and mealworm (Tables S1 and S2); lower magnification, SEM images of bryozoan chitin representing the Spider Web pattern (Figure S3a–c); TEM images of chitin nano bundles and nanocrystals from Bryozoa, sea spider and mealworm (Figures S3–S6); pitch sizes details of chitin nanocrystal and mealworm chitin nanocrystal film (Figure S8) (PDF)

## References

[1] Liu M., Wang S., Jiang L. (2017). Nature-inspired superwettability
systems. Nat. Rev. Mater..

[2] Wang H., Yang Y., Guo L. (2017). Nature-inspired electrochemical energy-storage
materials and devices. Adv. Energy Mater..

[3] Liu Y., He K., Chen G., Leow W. R., Chen X. (2017). Nature-inspired
structural
materials for flexible electronic devices. Chem.
Rev..

[4] Chang X., Feng Y., Guo B., Zhou D., Li L. (2022). Nature-inspired
micro/nanomotors. Nanoscale.

[5] Bouligand Y. (1972). Twisted fibrous arrangements in biological
materials
and cholesteric mesophases. Tissue Cell.

[6] Luo Y., Li Y., Liu K., Li L., Wen W., Ding S., Huang Y., Liu M., Zhou C., Luo B. (2023). Modulating
of bouligand structure and chirality constructed bionically based
on the self-assembly of chitin whiskers. Biomacromolecules.

[7] Kose O., Tran A., Lewis L., Hamad W. Y., MacLachlan M. J. (2019). Unwinding
a spiral of cellulose nanocrystals for stimuli-responsive stretchable
optics. Nat. Commun..

[8] Wang C., Tang C., Wang Y., Shen Y., Qi W., Zhang T., Su R., He Z. (2022). Chiral photonic materials
self-assembled by cellulose nanocrystals. Curr.
Opin. Solid State Mater. Sci..

[9] Chela-Flores J. (1994). The origin
of chirality in protein amino acids. Chirality.

[10] Grunenfelder L., Suksangpanya N., Salinas C., Milliron G., Yaraghi N., Herrera S., Evans-Lutterodt K., Nutt S., Zavattieri P., Kisailus D. (2014). Bio-inspired impact-resistant
composites. Acta Biomater..

[11] Wu K., Song Z., Zhang S., Ni Y., Cai S., Gong X., He L., Yu S.-H. (2020). Discontinuous fibrous
Bouligand architecture enabling formidable fracture resistance with
crack orientation insensitivity. Proc. Natl.
Acad. Sci. U. S. A..

[12] Ling S., Kaplan D. L., Buehler M. J. (2018). Nanofibrils
in nature and materials
engineering. Nat. Rev. Mater..

[13] Liu H., Feng Y., Cao X., Luo B., Liu M. (2021). Chitin nanocrystals as an eco-friendly and strong anisotropic
adhesive. ACS Appl. Mater. Interfaces.

[14] Narkevicius A., Parker R. M., Ferrer-Orri J., Parton T. G., Lu Z., van de Kerkhof G. T., Frka-Petesic B., Vignolini S. (2022). Revealing
the Structural Coloration of Self-Assembled Chitin Nanocrystal Films. Adv. Mater..

[15] Narkevicius A., Steiner L. M., Parker R. M., Ogawa Y., Frka-Petesic B., Vignolini S. (2019). Controlling the self-assembly behavior of aqueous chitin
nanocrystal suspensions. Biomacromolecules.

[16] Jung H.-S., Kim M. H., Park W. H. (2019). Preparation and structural investigation
of novel β-chitin nanocrystals from cuttlefish bone. ACS Biomater. Sci. Eng..

[17] Kaya M., Baublys V., Šatkauskienė I., Akyuz B., Bulut E., Tubelytė V. (2015). First chitin
extraction from Plumatella
repens (Bryozoa) with comparison to chitins of insect and fungal origin. Int. J. Biol. Macromol..

[18] Laumer C. E., Fernández R., Lemer S., Combosch D., Kocot K. M., Riesgo A., Andrade S. C., Sterrer W., So̷rensen M. V., Giribet G. (1906). Revisiting metazoan phylogeny with genomic sampling
of all phyla. Proc. R. Soc. B.

[19] Howard R. J., Giacomelli M., Lozano-Fernandez J., Edgecombe G. D., Fleming J. F., Kristensen R. M., Ma X., Olesen J., So̷rensen M. V., Thomsen P. F. (2022). The Ediacaran origin
of Ecdysozoa:
integrating fossil and phylogenomic data. J.
Geol. Soc..

[20] Wu Q., Mushi N. E., Berglund L. A. (2020). High-strength
nanostructured films
based on well-preserved α-chitin nanofibrils disintegrated from
insect cuticles. Biomacromolecules.

[21] Ifuku S., Nogi M., Yoshioka M., Morimoto M., Yano H., Saimoto H. (2010). Fibrillation of dried
chitin into 10–20 nm nanofibers
by a simple grinding method under acidic conditions. Carbohydr. Polym..

[22] Fan Y., Saito T., Isogai A. (2008). Preparation
of chitin nanofibers
from squid pen β-chitin by simple mechanical treatment under
acid conditions. Biomacromolecules.

[23] Kaya M., Mujtaba M., Ehrlich H., Salaberria A. M., Baran T., Amemiya C. T., Galli R., Akyuz L., Sargin I., Labidi J. (2017). On chemistry of γ-chitin. Carbohydr. Polym..

[24] Jang M. K., Kong B. G., Jeong Y. I., Lee C. H., Nah J. W. (2004). Physicochemical
characterization of α-chitin, β-chitin, and γ-chitin
separated from natural resources. J. Polym.
Sci., Part A: Polym. Chem..

[25] Zhang M., Li Y., Wang W., Yang Y., Shi X., Sun M., Hao Y., Li Y. (2020). Comparison of physicochemical and rheology properties
of Shiitake stipes-derived chitin nanocrystals and nanofibers. Carbohydr. Polym..

[26] Goodrich J. D., Winter W. T. (2007). α-Chitin nanocrystals
prepared from shrimp shells and their specific surface area measurement. Biomacromolecules.

[27] Onsager L. (1949). The effects
of shape on the interaction of colloidal particles. Ann. N.Y. Acad. Sci..

[28] Parton T. G., Parker R. M., van de
Kerkhof G. T., Narkevicius A., Haataja J. S., Frka-Petesic B., Vignolini S. (2022). Chiral self-assembly
of cellulose nanocrystals is driven by crystallite bundles. Nat. Commun..

[29] Střelcová Z., Kulhánek P., Friák M., Fabritius H.-O., Petrov M., Neugebauer J., Koča J. (2016). The structure
and dynamics of chitin nanofibrils in an aqueous environment revealed
by molecular dynamics simulations. RSC Adv..

