# Supplementary material for: Role of Invertebrate Biological Origin in Chitin Nanocrystal’s Morphology, Chirality, and Self-Assembly
Source: Langmuir. 2025 Jun 5;41(23):15004–13. doi: 10.1021/acs.langmuir.5c01167 (PMC12177918; doi:10.1021/acs.langmuir.5c01167)
Supplement: Supplementary file 1 [file la5c01167_si_001.pdf]

Supporting Information

Role of Invertebrate Biological Origin in Chitin Nanocrystal's Morphology, Chirality, and Self-Assembly

Murat Kaya[a,b*], Kui Yu[b,c,*], Kine Østnes Hansen[d], Mohammed Al-dubai[b], Martin Vinther Sørensen[e], Muhammad Mujtaba[f*]

[a] *Department of Molecular Biology and Genetics, Faculty of Science and Letters, Istanbul Technical University, Istanbul 34469, Turkey*
[b] *Yusuf Hamied Department of Chemistry, University of Cambridge, Lensfield Road, Cambridge, CB2 1EW UK*
[c] *Department of Bionanoscience Kavli Institute of Nanoscience Delft University of Technology Van der Maasweg 9, Delft 2629 HZ, The Netherlands*
[d] *Marbio, UiT–The Arctic University of Norway, Breivika, Tromsø, N-9037 Norway*
[e] *Natural History Museum of Denmark, University of Copenhagen, 2100 Copenhagen, Denmark*
[f] *VTT Technical Research Centre of Finland Ltd, P.O. Box 1000, Espoo, FI 02044, Finland*

***Email:** muhammad.mujtaba@vtt.fi (Muhammad Mujtaba); kayamurat@itu.edu.tr (Murat Kaya); k.yu-2@tudelft.nl (Kui Yu)

Table of Contents

1. **Table S1.** Thermal analyses result of chitin isolates and ChNCs from Bryozoa, sea spider and mealworm.
2. **Table S2:** Elemental analysis results of chitin isolates obtained from Bryozoa, sea spider and mealworm
3. **Table S3:** d-spacing and Miller Index (hkl) of XRD peaks.
4. **Figure S1:** Transmission electron microscopy (TEM) image of an ultra-thin section obtained via microtomy from a sea spider (Arthropoda). The image clearly displays the natural Bouligand structure characteristic of the cuticle organization.
5. **Figure S2.** Capillary tubes under strong polarized light containing ChNC suspensions from both Arthropoda (represented by Sea spider) and Bryozoa. Arthropoda-derived sample exhibits vivid coloration under polarized light, providing direct visual evidence of a fingerprint pattern in aqueous suspension. In contrast, the absence of such optical features in the Bryozoa-derived sample suggests a lack of long-range chiral nematic ordering at the dispersion stage.
6. **Figure S3.** Lower magnification, SEM images of bryozoan chitin representing the Voronoi Pattern.
7. **Figure S4.** TEM images of chitin nanobundles and nanofibers. a,b,c,d) from bryozoan chitin, e,f,g,h) seaspider chitin and I,j,k,l) mealworm chitin.
8. **Figure S5.** TEM images of chitin nanocrystals produced from Bryozoa.
9. **Figure S6.** TEM images of chitin nanocrystals produced from sea spider.
10. **Figure S7.** TEM images of chitin nanocrystals produced from mealworm.
11. **Figure S8.** Pitch sizes of sea spider chitin nanocrystal film (SChNCF) and mealworm chitin nanocrystal film (MChNCF).

TABLES

Table S1. Thermal analyses result of chitin isolates and ChNCs from Bryozoa, sea spider and mealworm.

	**Water content (between 25 – 150 °C) (%)**	**Maximum degradation temperature (°C)**	**Ash content (%)**
Bryozoa chitin	3.7	357.5	29.6
Sea spider chitin	1.6	378.5	20.1
Mealworm chitin	3.1	389	13.3
Bryozoa ChNC	4.8	351.8	23.1
Sea spider ChNC	7.1	353.3	21.5
Mealworm ChNC	3.9	311.7	30.8

Table S2: Elemental analysis results of chitin isolates obtained from Bryozoa, sea spider and mealworm

**Chitin isolates**	**%C**	**%H**	**%N**	**%DA**
Bryozoa chitin	43,79 ±0,07	6,35±0,01	6,41±0,03	98,35±1,06
Sea spider chitin	43,57±0,01	6,31±0,01	6,36±0,01	99,1±0,42
Mealworm chitin	43,79±0,02	6,37±0,07	6,63±0,02	85,3±0,66

Table S3: d-spacing and Miller Index (*hkl*) of XRD peaks.

**2θ (°)**	**d-spacing (Å)**	**Miller Index (hkl)**
9	~9.4	(020)
12	~6.8	(021)
19	~4.7	(110)
23	~3.7	(120)
26	~3.3	(130)

FIGURES

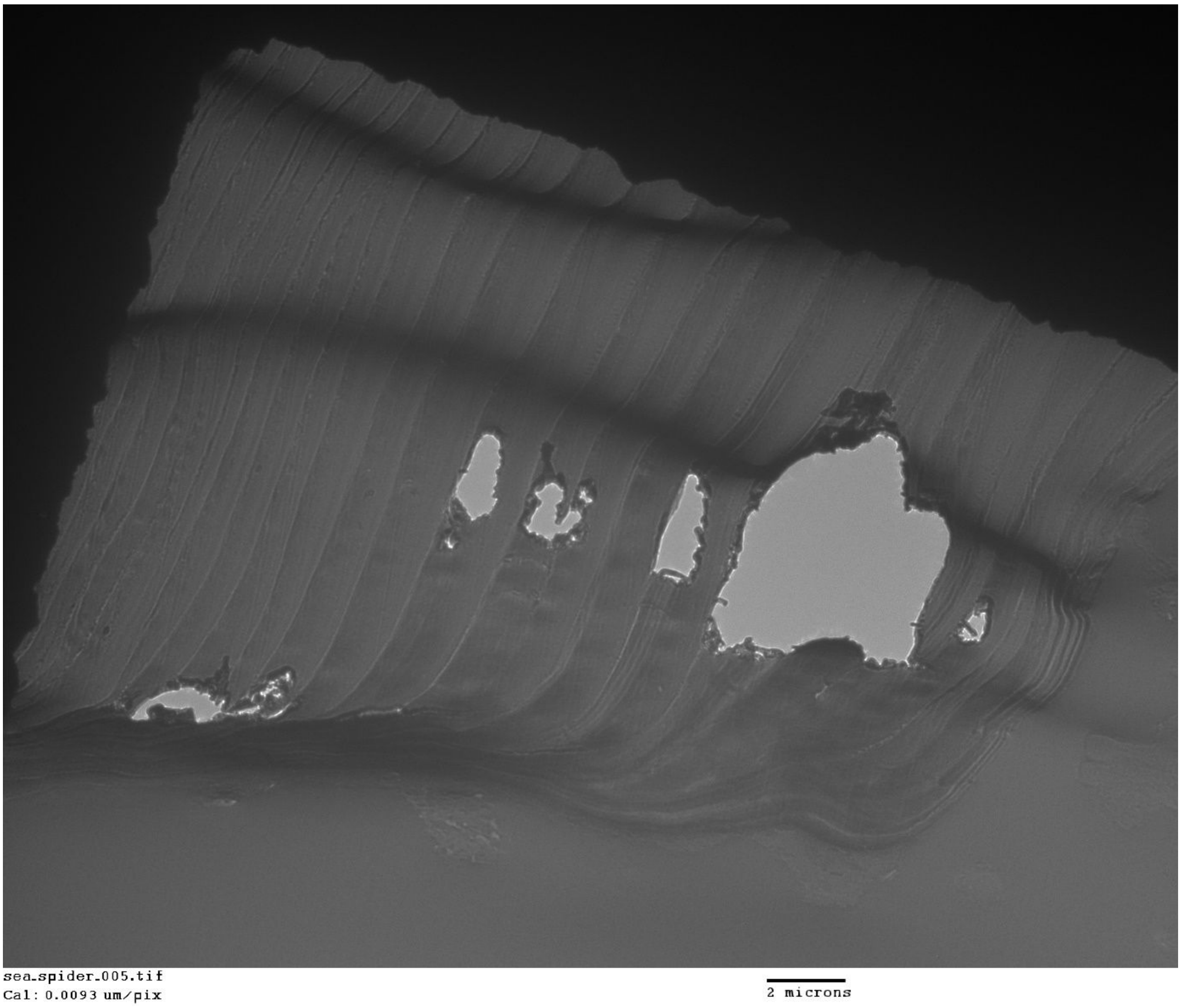

Figure S1: Transmission electron microscopy (TEM) image of an ultra-thin section obtained via microtomy from a sea spider (Arthropoda). The image clearly displays the natural Bouligand structure characteristic of the cuticle organization.

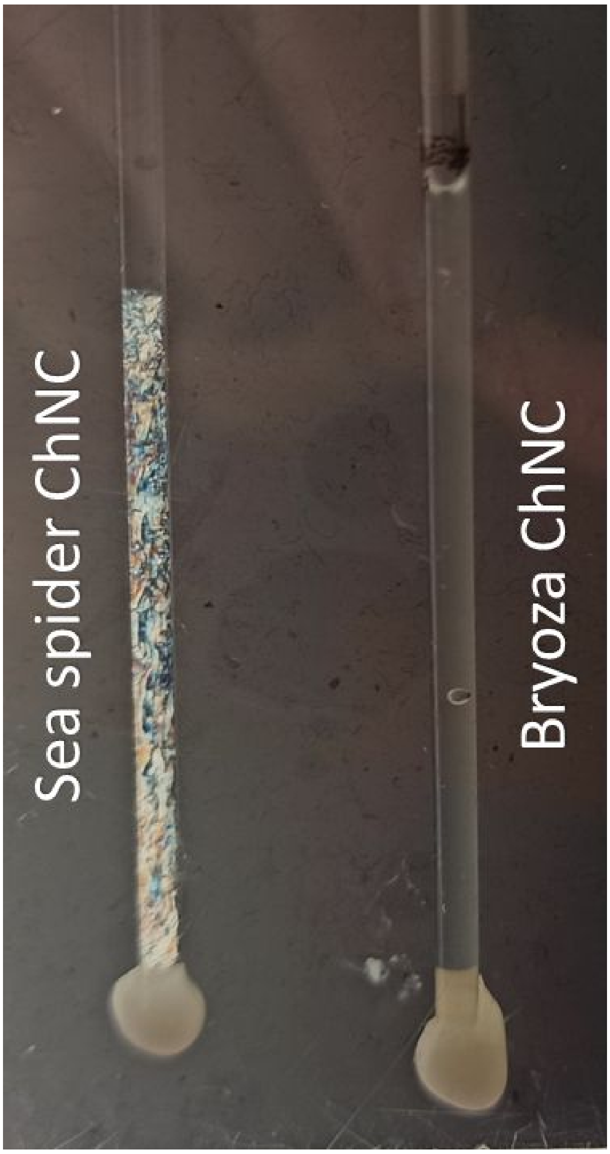

Figure S2. Capillary tubes under strong polarized light containing ChNC suspensions from both Arthropoda (represented by Sea spider) and Bryozoa. Arthropoda-derived sample exhibits vivid coloration under polarized light, providing direct visual evidence of a fingerprint pattern in aqueous suspension. In contrast, the absence of such optical features in the Bryozoa-derived sample suggests a lack of long-range chiral nematic ordering at the dispersion stage.

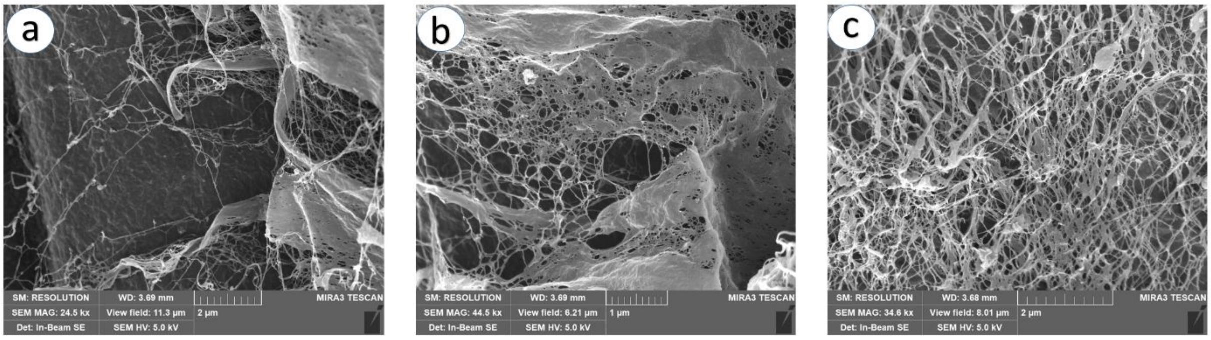

Figure S3. Lower magnification, SEM images of (a-c) bryozoan chitin representing the Voronoi Pattern.

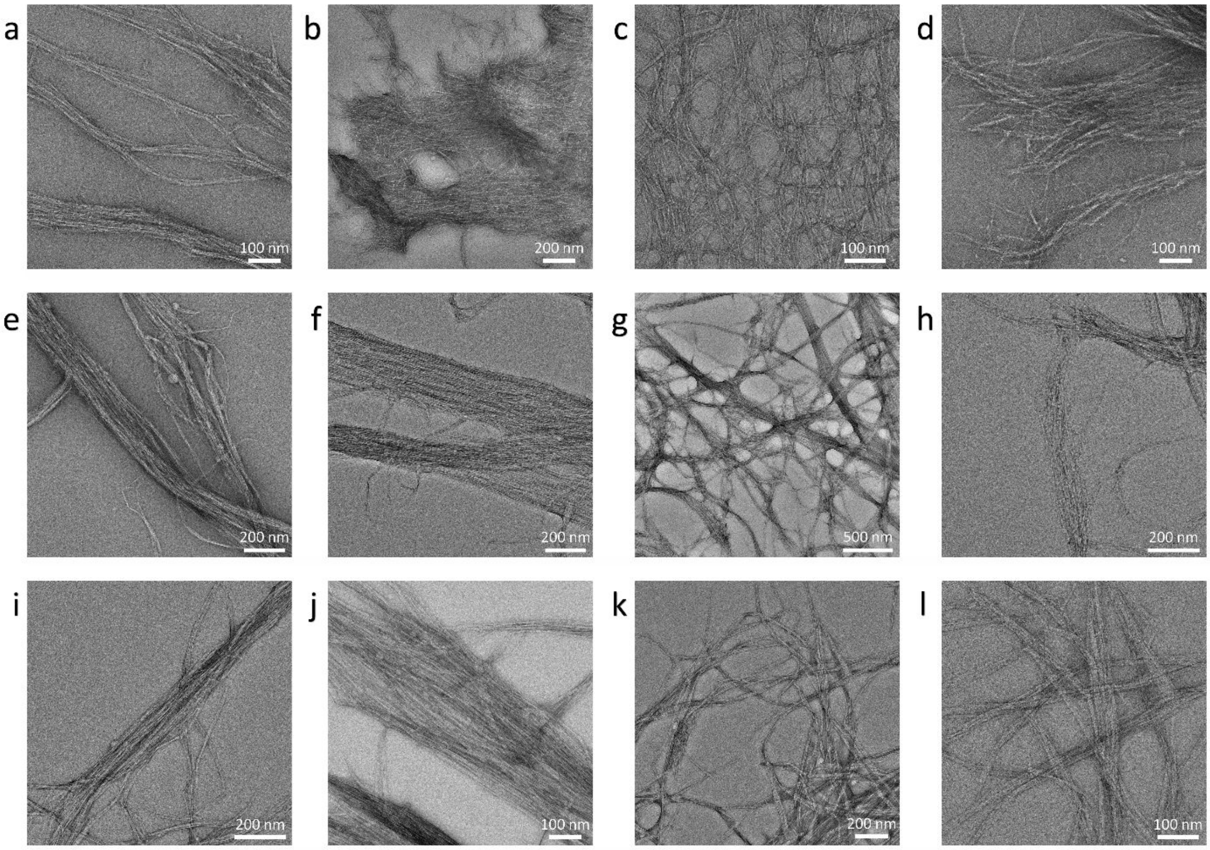

Figure S4. TEM images of chitin nanobundles and nanofibers. a,b,c,d) from bryozoan chitin, e,f,g,h) seaspider chitin and I,j,k,l) mealworm chitin.

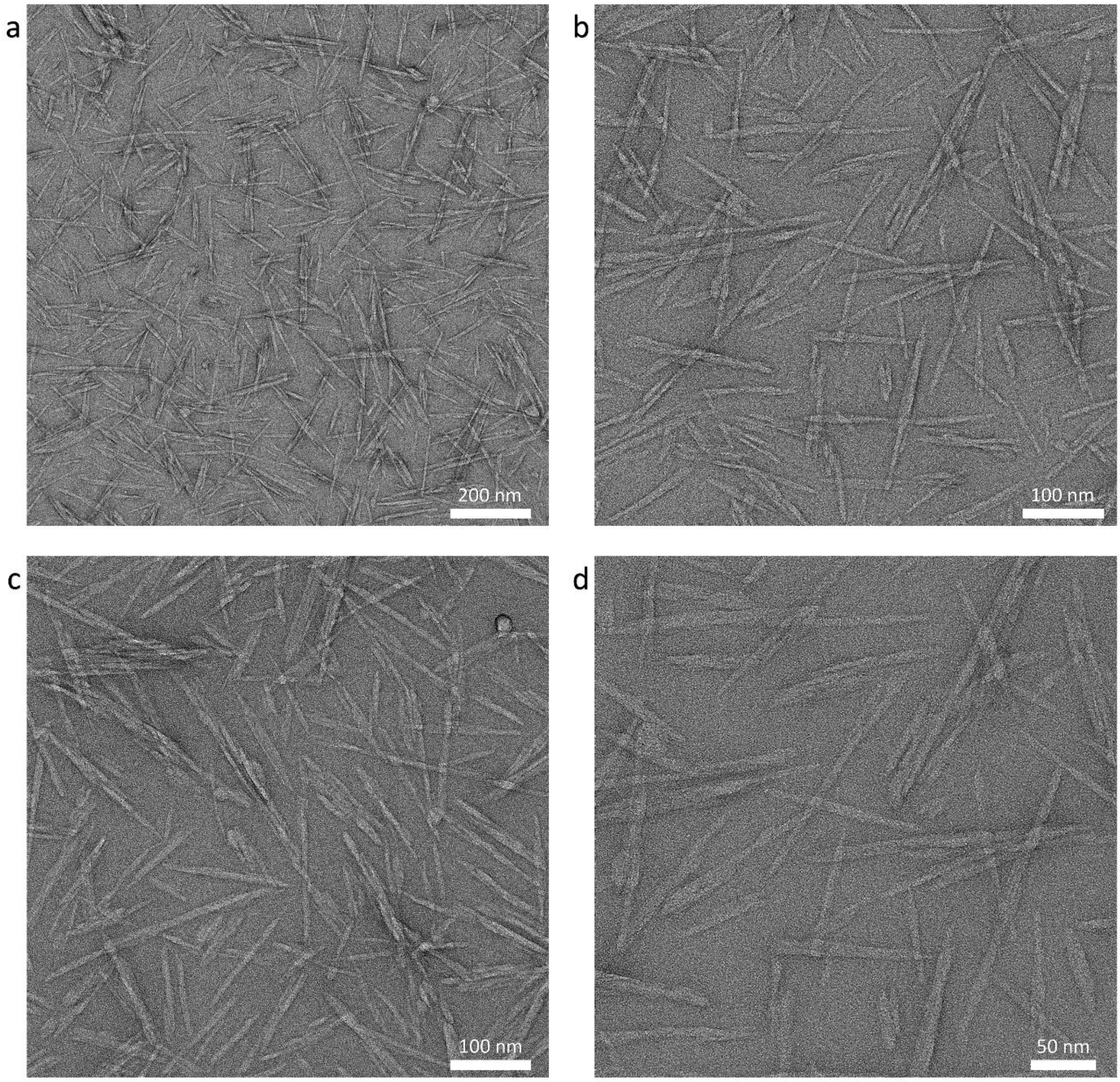

Figure S5. TEM images of (a-d) chitin nanocrystals produced from Bryozoa.

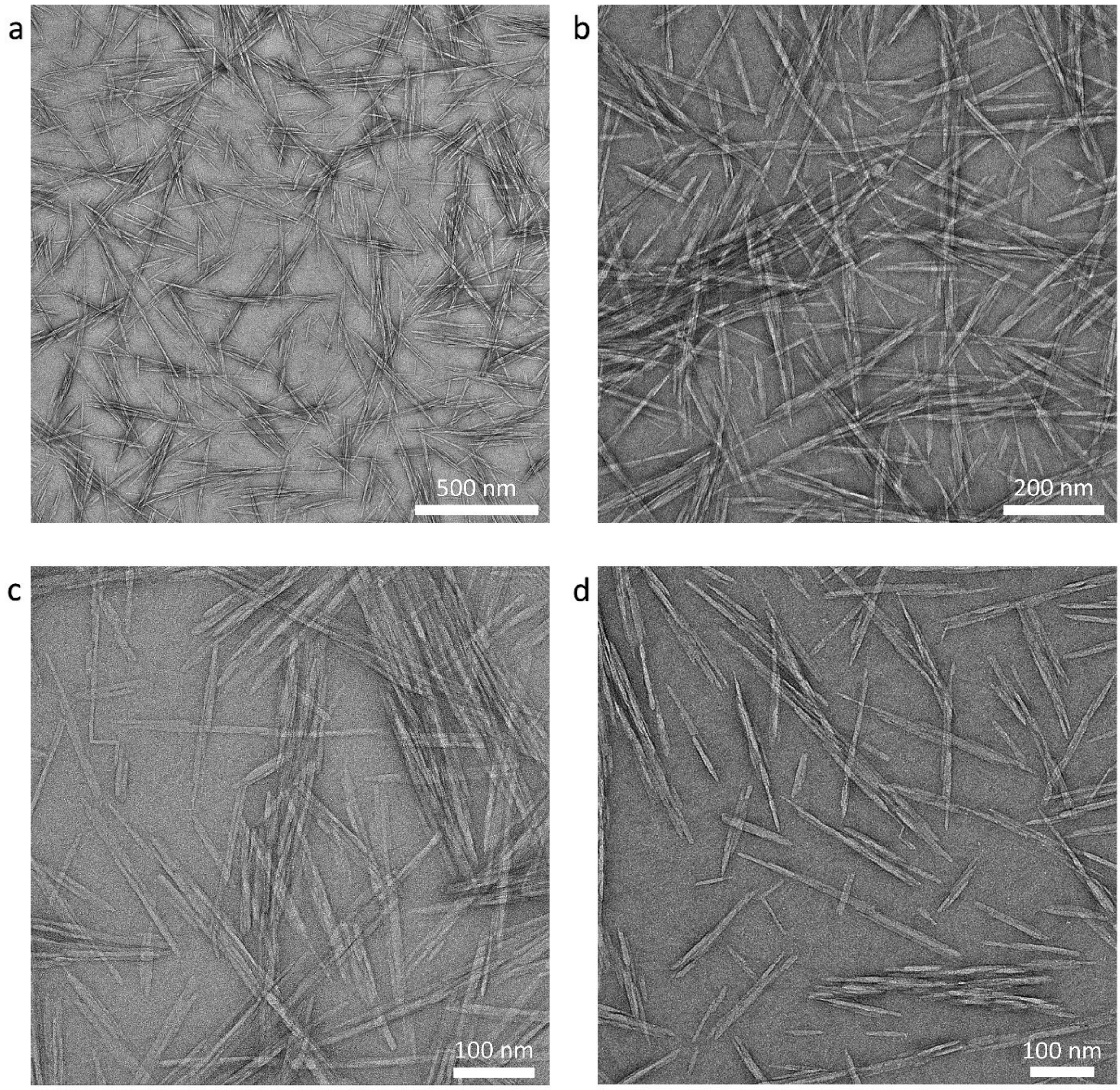

Figure S6. TEM images of (a-d) chitin nanocrystals produced from sea spider.

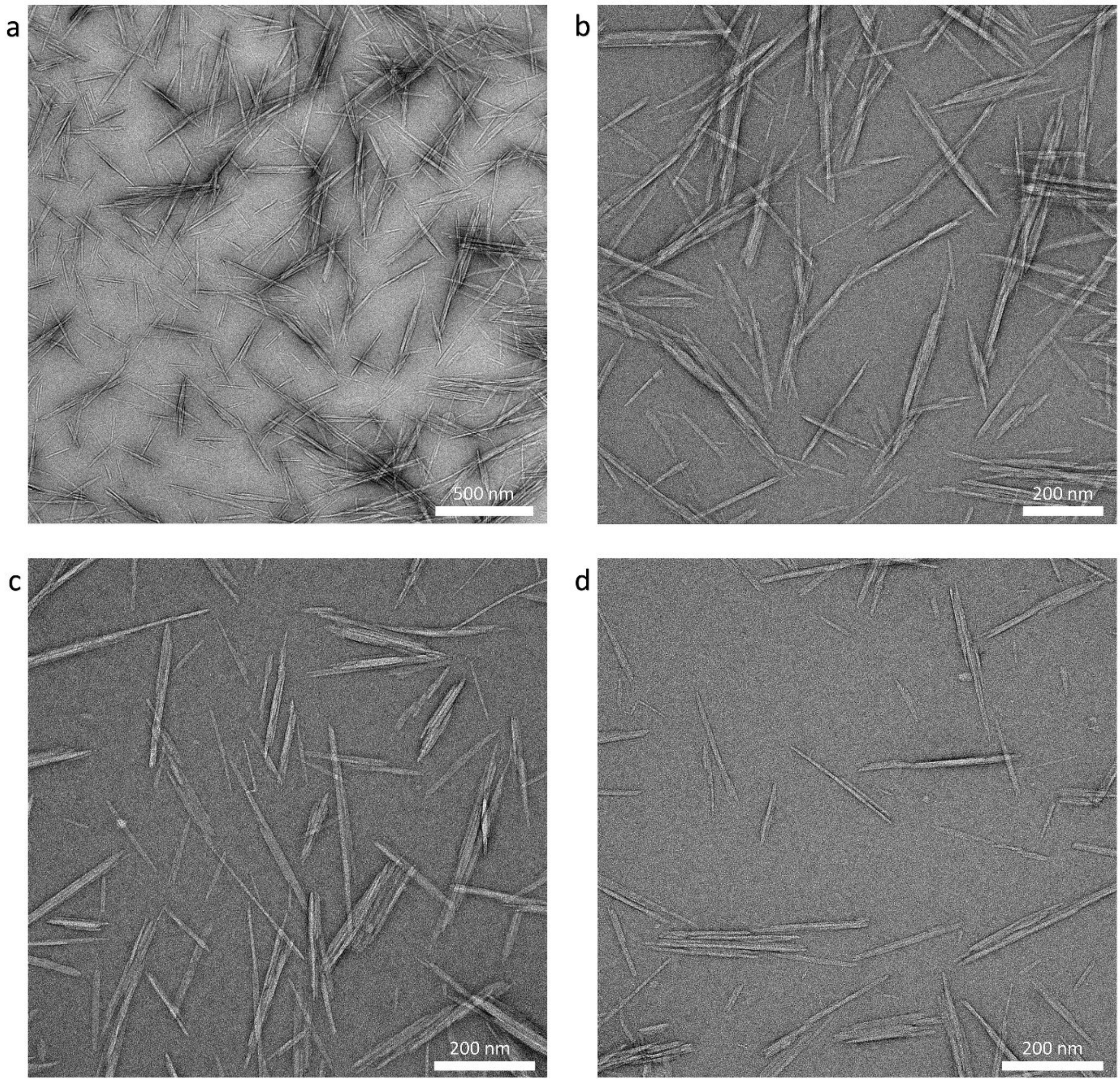

Figure S7. TEM images of (a-d) chitin nanocrystals produced from mealworm.

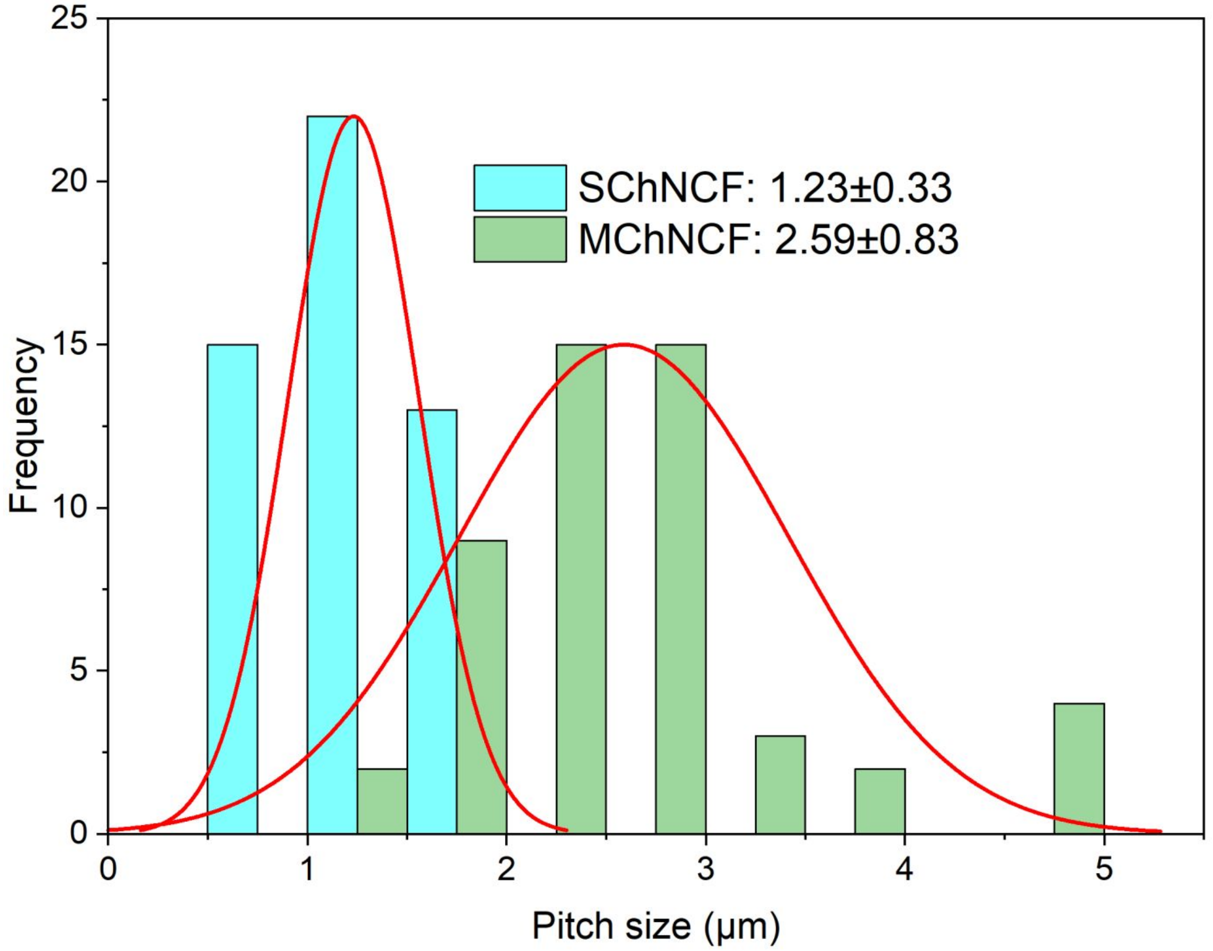

Figure S8. Pitch sizes of sea spider chitin nanocrystal film (SChNCF) and mealworm chitin nanocrystal film (MChNCF).
